# The Transactions of the American Medical Association, Vol. IX. 1856

**Published:** 1857-01

**Authors:** 


ART. IV.—*The Transactions of the American Medical Association.* Instituted 1847. Vol. IX. Philadelphia: Printed for the Association, by T. K. & P. G. Collins. 1856. 907 pages.

The first characteristic, apart from the comely style of paper and printing, which attracts our attention in the new volume of Transactions is its increased thickness. It numbers nearly a hundred and fifty pages more than its predecessor of last year, and contains over a hundred and fifty pages more of strictly scientific matter.

Whether this rapidly growing bulk be a subject of congratulation to the members generally must depend, as usual, upon the nature of the contents, which it is our task to analyze. With the prospect of such an undertaking, spread out in its colossal length and breadth before us, we can scarcely be regarded as unbiassed in our judgment on the question, and we shall therefore leave it as an open one, at least until our present duty is discharged.

The admirable address of the President, Professor George B. Wood, which occupies the first place in the regular *transactions*, is already before the profession. It has been widely read, and, we doubt not, correspondingly enjoyed and appreciated; and needs no additional discussion at our hands.

First among the reports appears the second part of Dr. Frank H. Hamilton's valuable papers on "Deformities after Fractures." This is considerably the longest article in the volume. We regard it as one of the most useful, and hence, most honorable to the Association. In the preparation of these two essays on a very important practical subject of inquiry, Dr. Hamilton has set an example which we hope may be often followed by the working members of the Association in relation to other no less important and still quite unsettled practical questions. As the investigations of Dr. Hamilton have grown into a "Treatise on Fractures and Dislocations," which the author is allowed to publish separately, after the completion of the report to the Association, we shall submit a very condensed outline of the portion now before us.

This begins with Chapter IV, an exceedingly interesting one, on Fractures of the Scapula; and considers, in successive chapters, fractures of the humerus, of the radius, of the carpus, of the metacarpal bones, and of the digital phalanges. We have been so much attracted by the details, and by the well-considered, yet always modestly-expressed, deductions of the indefatigable author, as to be unfitted for the work of rendering a connected abstract of his communication *in extenso*. The temptation is urgent to quote freely from the different chapters, but we must confine ourselves chiefly to figures and enumerations.

Three cases are narrated, and thirteen cabinet specimens described, of different fractures of the scapula. In relation to these fractures the views are cited of numerous authorities, among which we may name most of the leading surgical writers, past and present, as well as many of inferior note. Fracture separating the glenoid cavity from the body of the scapula is declared to be very rare, almost impossible. Fracture of the acromion process, also, is shown to be more rare than it was supposed to be; non-osseous union of the epiphysis being the real nature of the lesion which has been mistaken for a genuine fracture.

Sixty-seven cases are presented of fractures of the humerus, and nine different specimens are carefully described. These cases are also given in a table, from which it appears that there were of the upper third, 13; of the middle third, only 10; and of the lower third, 44: this result being at variance with the experience of Amesbury, Lizars, Bransby Cooper, and others, according to whom the fracture most frequently involves the middle third. A passage in this chapter has tempted us to quote it, on account of the important warning it conveys.

"Perhaps no place will be more appropriate than this to speak of the difficulty of diagnosis in fractures about the joints, and especially in fractures occurring in the vicinity of the shoulder-joint: a difficulty so serious as materially to embarrass the surgeon in his prognosis; and which, it must certainly not be denied, diminishes the value of my own conclusions, as based upon my recorded cases. It is only, after all, by an examination of a great number of cases, both before and after death, that we shall ever arrive at a complete solution of these difficult questions. To this point already the labors of Sir Astley Cooper, R. Smith, Key, and others, have been especially directed; yet the constant mistakes committed to-day by the most experienced surgeons, not to speak of those acknowledged by Sir Astley himself, testify to the imperfection of our knowledge." P. 129.

Professor Hamilton prefers in the treatment of fractures of the humerus "a broad and thick splint of gutta percha, sufficiently long to extend from the neck to the wrist, moulded accurately, and applied to the shoulder, arm, and forearm, while the limb is flexed to a right angle, and while extension is being made upon the humerus. This being properly padded and secured in place by rollers, I place the arm in a sling beside the body. The sling must, however, be so arranged by being looped under the wrist, and not under the elbow, as that the weight of the elbow and lower part of the arm may aid in making extension." P. 136.

Thirty-eight original cases and three cabinet specimens are given, with a table, in the chapter on fractures of the radius. Of the cases of fractured radius reported, three were supposed to be of the neck of the bone, but not insisted on, owing to the admitted difficulty of the diagnosis and the rareness of such cases; two were of the middle third; thirty-three were of the lower third, twenty-six of these last being "Colles' fracture." "Barton's fracture" does not appear in the list, as the author has never met with one, either in practice or in cabinet preparations, and, consequently, regards it as an extremely rare form of fracture.

The deformities which may occur in fractures of this bone are fully and thoroughly discussed, and very forcibly as well as usefully pointed out. The treatment of fractures involving the lower end of the radius, necessarily receives a large share of attention. The various modifications and applications of the "adduction" method, and the uses and properties of the pistol-shaped splint of Cline and Dupuytren, and their successors, down to R. Smith, Nelaton, Bond and others, are carefully considered. He recommends the splint of Bond, or an improvement of it by Dr. E. P. Smith, of Buffalo; but in his own practice habitually employs a simpler form (described on p. 176), which closely resembles the extemporaneous contrivance of Dr. Hays, although somewhat neater and more permanent.

Fractures of the ulna are typified in twenty-two cases and one cabinet specimen, and the usual tables. Of these fractures, eleven were in the upper third; five in the middle, and six in the lower third. Forty cases are recorded and tabulated of fractures of both radius and ulna, of which one occurred through the upper third, fourteen through the middle, and twenty-five through the lower.

The danger of mortification, more frequent in these fractures than in others, is well described, and the idea suggested that it may not be invariably due to mismanagement of the surgeon. The use and disuse of inner bandages, graduated compresses, narrow and yielding splints, in contradistinction to the employment of outside bandages only, with broad padded splints of wood or other firm material, instead of pasteboard, are instructively discussed. Of the different positions of the forearm, the supine, which is advocated by Lonsdale, South, Nelaton and others, is adopted by the reporter.

No cases are reported of fractured carpus. Of metacarpal fractures there are seven cases, one being of the thumb, two of the forefinger, and four of the little finger. Finally, we have fifteen cases of fractures of the phalanges. In conclusion, it should be stated that the report is conveniently illustrated by four lithographic plates, in which specimens and apparatus are very distinctly represented.

The second article is a very important and deeply interesting report by the Committee on Hydrophobia, of which Dr. Blatchford, of New York, was chairman. It presents a statistical table, compiled from various sources, of one hundred and six cases recorded as hydrophobia occurring in the United States.

Of these, seventy-two terminated fatally. In eighty-nine the attack followed the bite of a dog; in nine, that of a cat; in one, of a raccoon; and in one, of a fox. According to records in England, France and the United States, of two hundred and fifty cases, the greatest number, seventy-eight, were inoculated in the spring months; sixty-five in December, January and February; fifty-nine in summer, and forty-eight in autumn.

Forty-one of the cases are new, having never been upon record before; and these are given, mostly, in full detail. The average period of incubation in eighty-nine cases, was seventy days; the average duration of the fatal cases was three days.

Several exceedingly interesting facts are given in regard to the history of the disease. It is proved to be of comparatively frequent occurrence in France and Northern Europe, as well as in the United States, but rare in tropical climates. So little difference in the number of cases has been observed at different times of the year, that the committee conclude thermal influence to have no relation to its causation. This is, perhaps, the most important conclusion of the report. "Every investigation," say they (p. 250), "anywhere made, only proves that a belief in the influence of the 'dog star,' or climate or season of the year, as inducing or favoring the production of rabies, is an utter fallacy." Hence they stigmatize laws based upon such an hypothesis as "not only absurd, and their execution expensive and cruel, but positively injurious to the community," by creating a false security during the other seasons of the year.

Examining the hypotheses upon the etiology of the disorder, it is said (p. 240), "that putrid meat, or want of food and drink, never generates the disease in the dog, has been satisfactorily proved by experiment." It has, in fact, never been shown to have occurred directly from any cause but the *bite of a rabid or enraged animal.* All the animals susceptible to the disease are similar in the possession of teeth fitted for seizing and tearing their prey; they are all carnivorous, and "it is probable," say the committee, "that their peculiar liability to rabies consists in a *constitutional irascibility.*" Dogs fed on vegetable food, it would seem from the experience of the West India Islands, hardly ever become mad. After pointing out the resemblance between the manifestations of ordinary rage, and those of the disease, the committee say in conclusion (p. 243), "If, then, an enraged dog exhibits so many of the symptoms of rabies, especially the viscid frothy saliva so characteristic of the disease, and which all admit contains the virus, it is reasonable to conclude that the functional condition of the organs implicated in the disease, the very organs in which it is supposed the virus is generated, is in both cases very similar, and, therefore, capable of generating the virus. That the bite of such an animal should be followed by rabies is a most rational conclusion, and if such be the case, it is probably the *only* way the disease ever originated." In support of this conclusion the committee refer to several of their cases, and cite various authorities.

The manner in which this singular virus operates is left in mystery. The fact, however, that "the disease has been prevented by excision of the cicatrice after it had become painful, swollen, and discolored, a group of symptoms known by the term 'recrudescence,' and always followed by the disease under any other treatment, is positive proof that the virus is not directly absorbed into the system." On this account a number of very high authorities advise and urge the excision of the bitten parts, "*at any time before* the occurrence of the second inflammation," as an almost or quite certain prevention of hydrophobia. Among these is Mr. D. Blane, of London, who, upon the basis of long observation, asserts (p. 246), "that it is of no consequence that the excision of the part should be immediately effected;" but, as secondary inflammation may come on at any time, he advises to have it done as soon as convenient. This opinion is certainly momentous, if justified, as it appears to be, by many data brought forward by the committee. Although still in a great measure speculative upon all decisive points, and inconveniently diffuse in style, this report does great credit to the ability and untiring industry of the committee. It is full of interesting and curious information, as well practical as theoretical, and will repay an attentive perusal.

The report on Hydrophobia is followed by two papers in succession, each of which may be regarded in different quarters, as advocating views and tendencies of much greater moment than the study of the disorder which had just been honored with so large a portion of the volume. They have already attracted towards their several propositions an amount of attention which is not likely to flag, now that the whole debate is published in its official form. They are both able and earnest arguments on vitally important questions; and as such are entitled to the careful examination of every member of our profession. As, however, their adequate discussion would involve more space than can be allowed us, we prefer commending them at length to the especial attention of our readers, to any attempt at analytical *exposé* of their pith and purpose merely.

The first in order is the Report of Dr. S. D. Gross on the "Causes which Impede the Progress of American Medical Literature;" the second is the regular Report of the Committee on Medical Literature, by their chairman, Dr. R. J. Breckinridge, of Louisville, Kentucky.

The essential part of the Report on Plans of Organization for State and County Medical Societies (which follows next, and of which the chairman was Dr. A. B. Palmer, of Detroit, Michigan), is contained in the following extract from a series of resolutions proposed at its close:

"2. *Resolved*, That this Association earnestly recommend to all County Medical Societies to incorporate into their constitutions or by-laws, provisions for the election of a Board of Censors, whose duty it shall be to examine all persons who may apply for admission into the office of any member of the society as students of medicine; and also to incorporate provisions to prevent any member of such society from admitting, as a student, any person who shall not first receive from the Board of Censors a certificate of a good moral and intellectual character, a good English education, including a thorough knowledge of the English language, and a respectable acquaintance with its literature, and with the art of composition, a fair knowledge of the natural sciences, and, at least, the more elementary mathematics, including the chief elements of algebra and geometry, and such a knowledge of the ancient languages as will enable him to read current prescriptions, and appreciate the technical language of the natural sciences and of medicine.

"3. *Resolved*, That this Association also earnestly recommend to local or County Societies to incorporate into their constitutions or by-laws provisions for making it the duty of each of their members to keep at least a brief record of all cases occurring in his practice, depending upon endemic or general causes, and report at least annually to a committee of the society to which he belongs, the number or percentage of different diseases occurring during each month, together with the particular type of each disease, the chief modifying circumstances under which it occurred, the general plan of treatment, and the results of the cases; and also that these societies make provision for the election of such committee, whose duty it shall be to receive and collate such reports, arranging them in due form, and adding such remarks as may assist to their proper understanding, and to transmit them annually, thus analyzed, to a committee of the State Society to which the local or County Society shall be auxiliary; and this Association further recommends that the State Societies make provision in their constitutions or by-laws for the appointment of a committee, whose duty it shall be to receive such reports from the local or County Societies to again arrange them with other reports from similar societies, placing them in a condensed or tabulated form, and report them annually, with proper remarks, to a committee of this Association, to which the State Societies are recommended to become auxiliary." Pp. 409, 410.

The report closes with a plan of constitution for State Societies.

The report by Dr. N. S. Davis, of Chicago, which follows the above, "On the Changes in the Composition and Properties of the Milk of the Human Female, produced by Menstruation and Pregnancy," details the results of the microscopic and chemical examination of the milk of three healthy women during the three periods of simple lactation, lactation with pregnancy, and lactation with menstruation.

It is inferred, so far as may be from these limited observations, that:

"1st. The occurrence of pregnancy during lactation produces very marked diminution of all the solid or nutritive constituents of the milk, such diminution continuing to increase as the pregnancy advances.

"2d. In examining the separate proximate constituents, it will be observed that a much greater relative diminution takes place in the casein, the butter or oil, and the salts, than in the sugar and extractive matter.

"3d. There appears to be added to the milk secreted during the progress of utero-gestation some of the *granular* bodies or colostrum corpuscles, and numerous infusoria or animalcular germs, which have been very rarely found in healthy milk.

"4th. Changes the same in kind take place in the milk secreted after the establishment of regular menstruation, but much less in degree; and the relative diminution of the several constituents is uniform." Pp. 424, 425.

After some remarks upon the changes in the qualities of the milk, as inferred partly from the ascertained changes in its composition, and partly from direct observation of its effect upon the nursing infant, the reporter terminates his paper for the present by reminding the Association that these investigations "require much time and labor, though the results may be stated in a few words or figures." "The important practical bearing," he further says, "of the results obtained, thus far, will be obvious to every intelligent physician. But the examinations and analyses, microscopic and chemical, should be multiplied until they are sufficient to render all conclusions drawn from them demonstrated truths."

The Committee "On the Sanitary Police of Cities" (Dr. J. M. Newman, of Buffalo, Chairman), observes that, "in view of what has already been accomplished by previous committees, the range of subjects treated in this report will necessarily be much more circumscribed than would otherwise be proper with a subject so broad."

Emphasis is laid in the report upon the predominance of *preventable* disease, especially in cities; which, as they have oftentimes been called plague-spots in morals and politics, so are they often plague-spots of a verity, and the most favorite haunts for the ravages of epidemics.

Interesting and important tables are given (pp. 434, 435) of the rates of *deaths* to *population*, in New York, Philadelphia, Boston, Providence, Baltimore, Charleston, Chicago, and New Orleans, for which we must recommend the report to the attention of our readers. A statement is given, also, of the proportion of deaths from "zymotics" in the United States at large, and in various special localities; varying from 20 to over 50 per cent. of the whole number of deaths. In the class of zymotics are named cholera, cholera infantum, croup, diarrhœa, dysentery, erysipelas, intermittent and remittent fevers, typhus, hooping-cough, influenza, measles, scarlatina, small-pox, syphilis, thrush, cholera morbus, typhoid fever, and yellow fever. *Puerperal fever*, which might have been added with propriety, is not named.

In regard to cholera, "emphatically an epidemic of cities," it is remarked that "there is an absence of a continuous, systematic course of remedial measures calculated to remove at all times the fruitful soil in which the pestilence finds root, and developes itself into a full harvest." "We should remember that the circle of its influence may radiate far from the centre of its birth." In the mortality statistics of the census of 1850, we find that the total number of deaths by "*fever*" of all varieties was 33,344; a greater number than by cholera, although that disease also was epidemic.

Upon the subject of "water supply," the committee remarks: "So long as the matter of supplying our towns with water is left in the hands of companies, or individuals, just so long will the evils resulting from an insufficient supply of this element be only partially remedied; the sources of disease, and the class of persons most in need, will never be reached." "This principle should be recognized, and no town or city should hereafter permit this right to pass from their control. The municipal corporation should rightfully be the means of dispensing health and happiness among the citizens, without the debasing question of profit, entering into every consideration."

Upon the subject of *Ventilation*, in dwellings and public edifices, a similar suggestion is made: "*that the evils are to be corrected, and to be corrected alone, by the strong arm of the law.*" "We want a *strong municipal law*, which shall plainly and distinctly say, in what manner our edifices shall be built," &c. &c. (P. 453.) "We have an approach to such a law, in the authority vested in our Boards of Health, to enter, in times of epidemics, and thin out an over-populated house. But, as prevention is better than cure, the evil should be prevented, not simply corrected."

As to *Sewerage*,—"the superintendence of the construction of all sewers, lateral as well as main, should be, it is urged, "*confided to the charge of the health officers of the town.*"

Attention is called particularly, to the subject of the influence exerted upon the health of a city, by the upturning and removal of the natural soil, especially in times of epidemics. The evidence of Dr. Barton, in regard to yellow fever, in New Orleans; of Dr. Hamilton, as to the outbreak of cholera, in Buffalo, in 1852, and of Dr. Newman himself, in regard to a similar epidemic, at Niagara, in 1854, is adduced. It is inferred, that "the emanations of such exposures undoubtedly have the property, under the influence of heat and a humid atmosphere, of giving the most fatal intensity to that form of disease, which may be determined by the epidemic constitution of the year."

*Compulsory Vaccination*, to insure that of the whole population, is recommended as the only effectual security against the ravages of small-pox.

In conclusion, the chairman boldly contends, "that to the physician belong the posts of honor and of profit conferred by the constitution of any sanitary commission, board of health, or any other office which is designed to devise and execute hygienic laws." "Let men be selected from the ranks of the profession for these posts, who will bring to the exercise of their duties a laudable professional ambition, and work from a love for their labor, and be actuated by the true spirit of the sanitary physician."

In Dr. A. J. Fuller's report *On the Treatment of Cholera Infantum*, we have an unpretending sketch of the pathology of the disease, followed by a succinct account of its treatment, upon principles recognized by most practitioners. There is little novelty in this paper, which is clearly written, and with a judicious brevity.

Dr. Horace Green has contributed a report on the *Use and Effect of Applications of Nitrate of Silver to the Throat, either in Local or General Disease.* The opening remarks exhibit the tendency of the paper, as follows:

> "Many years ago the celebrated Abernethy published to the world his treatise 'On the Constitutional Origin of Local Diseases.' His views have been pronounced enlightened and philosophical; and perhaps justly so. Certainly they were eminently suggestive, for they contributed more than those of any other writer of that period, to awaken among both surgeons and physicians a spirit of enlightened inquiry with regard to primary diseased action.
>
> "But the great work needed now more than any other, by both branches of the profession, and which might be termed, perhaps, the converse of that of Mr. Abernethy, is one which would embrace a full, enlightened, and philosophical history of the *Local Origin of Constitutional Diseases.* Until this neglected portion of the history of Medicine shall have been written by some second Abernethy, thoroughly enlightened and imbued with the vast importance of his subject, the value of topical medication, its effects and utility, cannot be fully appreciated by the profession."

Allusion is made to several monographs, in which the topical use of nitrate of silver is mentioned; as that of Dr. Mackness, of London, Dr. Hastings, Dr. Scott Alison, and Dr. Eben Watson, of Glasgow. Two other British writers are asserted to have borrowed largely, without acknowledgment, from Dr. Green's own work on the Diseases of the Air-Passages, published in 1846.

Different opinions are entertained upon the *rationale* of the "argentine" topical medication. Dr. Watson believes the nitrate of silver to be a local stimulant, and therefore useful only in a subacute or chronic inflammation.

Dr. Bennett, of Edinburgh, on the other hand, believes its action to be that of a "calmative or sedative." Dr. Scott Alison considers it to be an irritant to an acutely inflamed part, but, to one chronically inflamed, a beneficial tonic and stimulant. Others view its action as essentially protective, by the white coating produced by the application.

Remarks are made by the reporter upon the "effect of nitrate of silver in the treatment of follicular pharyngo-laryngeal disease;" in "acute and chronic laryngitis;" in "membranous croup;" in "œdema of the glottis;" in "hooping-cough;" in "spasmodic asthma;" and in "tuberculosis, following or complicated with bronchial inflammation."

The application of the sponge to the mucous membrane of the larynx, at least, is of course insisted on in these affections. A very interesting *resumé* is afforded upon the subject, in a manner less controversial than might have been anticipated from the position of the author in regard to it.

In membranous croup, Dr. Watson considers that it is the *pre-exudative* stage to which this local treatment is applicable. Others, especially in this country, have had very good success with it in later stages. Dr. Ware, of Boston, is quoted among these.

The first application of solutions of nitrate of silver to the air-passages in *hooping-cough*, is credited to Dr. Watson, of Glasgow; suggested, however, by the work of Dr. Green. In 167 cases of this disease, under the care of Dr. Watson and M. Joubert, 96 cases, more than half, were cured in two weeks; 61 cases in three or four weeks; 9 resisted the treatment, and 1 died. Under more ordinary treatment, the average duration, according to Williams, Copland, Walshe, West, and others, is from one and a half to three and a half months.

In spasmodic asthma, the treatment is urged on the ground of a somewhat peculiar view of its pathology. "The state of the larynx," says Dr. Watson, "in spasmodic asthma, has not hitherto received adequate attention, either from pathologists or physicians; in this opinion he expresses himself fully confirmed, that morbid contraction of the larynx is a frequent cause of the disease, and that a spasm of the glottis dependent upon a lesion of this organ, constitutes an essential part of a fit of asthma."

Where tubercular disease of the lungs is threatened or suspected, several authorities recommend the topical medication; for instance, it is adopted by Dr. Hastings (Treatise on Diseases of the Larynx and Trachea), Professor Watson, Dr. Cotton, Professor R. B. Todd, of King's College Hospital, and Professor Bennett, of Edinburgh.

The report of Dr. Green closes with a brief allusion to what he believes to be the successful and beneficial employment of "*catheterism* of the air-passages, or the injection of a solution of the nitrate of silver into the bronchial divisions,"—in one hundred cases of thoracic disease, of which, seventy-one are recorded as tubercular.

The report terminates with a reiteration of its author's well-known predilection for "this method of treating disease," whether in "bronchial affections" or in commencing tuberculosis.

Next follows the report, by Joshua B. Flint, M.D., "On the Best Mode of rendering the Patronage of the National Government tributary to the Honor and Improvement of the Profession." It commences with an eloquent protest against the "gross disregard of the honor and rightful prerogatives of the medical profession, in the practice of issuing patents to the proprietors of quack medicines." We cannot do justice to this part of the paper by an abstract. "It is gratifying to learn, however, by a letter from the Commissioner, that the practice of the office has been such, of late years, as to discourage this objectionable employment of its powers."

The absence of appropriations by Government to encourage or assist medical science in this country, is alluded to, and made the occasion of an "episode" upon medical literature in general, and the "American Medical Society in Paris" in particular.

The subject of the solicited pecuniary grant to Dr. Morton, for the origination of Etherization, is discussed, with little or no regret that it was not awarded.

The national debt of gratitude, it is contended, to the profession, might be best expressed by laying *upon the altar of the American Medical Association*, an appropriate tribute of acknowledgment. For annual prizes, and for the promotion of statistical and scientific inquiries, a considerable sum might be thus advantageously employed, which would never be missed from the public treasury. Analogous examples of such patronage are cited, as in the cases of Espy, Morse, and the heirs of Robert Fulton.

Further remarks are made upon the more direct relation of the Government to such members of the profession as are eligible to appointments connected with its official service; as in the army and navy. A degree of illiberality is here still a subject of complaint.

The Report on Medical Education, by W. H. Anderson, M.D., contains, with the oft-repeated, but no less correct sentiments, upon that subject, briefly and clearly expressed, at least one novel suggestion,—that "the courses of lectures attended by the student, should be left to the close of his term of study; and, that to facilitate his earlier progress, besides thorough office instruction, another book is needed: a book which will contain, in one, or at most, two volumes, dissertations on the various subjects which enter into the course of medicine."

There may be, we suspect, in the profession, very different opinions in regard to the desirableness of such a work.

A report "in part," on the "Medical Topography of the Eastern Shore of Maryland," is given by Dr. D. P. Wroth. It contains much interesting information, in respect to a long-settled and important region.

The "History of the Epidemic of Yellow Fever, in Charleston, S. C., in 1854," is furnished in an excellent paper, by Dr. D. J. Cain, who argues decidedly in favor of the *importation* and *contagiousness* of the disease.

Dr. Turner, of New Orleans, in his "Report on the Epidemics of Louisiana, Mississippi, Arkansas, and Texas," urges the view that yellow fever originates spontaneously, in New Orleans at least, not being, generally, "infectious;" but that, *under favorable circumstances*, it may be transported from one place to another, and even become communicable from person to person. This is the doctrine of contingent contagion.

So far as concerns the local origin of this disease in New Orleans, the documents connected with this report, as well as its own facts, and those of the well-known report of Dr. E. H. Barton, "On the Meteorology, Mortality, and Sanitary Condition of New Orleans, for 1854 and 1855," make an exceedingly strong case. The last-mentioned paper is of especial value in this regard, from its fulness of detail, and carefulness of preparation; and from its numerous and striking illustrations by diagrams and tables. Between these reports is placed a short, but interesting account, from personal observation, by Dr. Fenner, of the Epidemic of Yellow Fever at Norfolk and Portsmouth, Virginia, in 1855.

A brief paper follows, upon the physiological effects and chemical detection of Strychnia, by Lewis H. Steiner, M.D., of Baltimore. This is succeeded by one of still less length, by Dr. G. S. Palmer, entitled "A Partial Report upon a Uniform System of Registration of Births, Marriages, and Deaths, and the Causes of Death."

The Prize Essay for the year, by Dr. Henry Hartshorne, of Philadelphia, is a paper of about sixty pages, "On the Arterial Circulation; its Physiology, and Chief Pathological Relations." Although a proper effort to maintain impartiality is manifested, the opinions of the author are obviously decided; and appear to be opposed to those of a majority of modern writers upon the subject. The boldness of this difference is entitled to respect, although it may be viewed as temerity, or otherwise, according to the estimate placed by different readers upon the force of the reasons adduced for it.

The argument is chiefly drawn from 1. Histological induction, in regard to the probable nature of the activity of the arterial muscular coat; 2. Analogy, from comparison of the circulation of other animals, of different types; 3. Observation of accidental and pathological deviations.

The histological and analogical reasoning of the essay may be summed up in the following passages:

"Wherever it has been possible fully to study muscular action, the best of authorities agree in finding *no instance of active dilatation*, or *dynamic elongation of muscular fibre.* Hence, to suppose such a phenomenon in the *vascular system*, which, more than almost any other, exhibits facts only under the greatest obscurity and difficulty of interpretation, is to suppose what is *unphilosophical;* and, therefore, must be considered untenable or untrue."

"It may, very probably, be true, that in the simplest animals, as the protozoa, radiata, and lower mollusca, there may be no other determining cause for the slight variations which occur, than such as exist in plants, aided by the general animal contractility. But, when specialization begins, we find it at first shown in the creation of additional contractile vessels, which, as they differ in magnitude, number, and force, must vary the supply of fluid which they afford. And, when the specialization approaches its highest point, the irritability of vascular trunks is no longer entirely controlled by their direct stimuli, but is confided, to some extent, to a nervous apparatus; as is the case with all the muscles of the organic, hardly less than of the voluntary system."

The experiments of the Webers and others, are believed, by the essayist, to have been made too hastily the basis of the opinion, that the arteries contract only with a tonic rigidity, and not with an alternating or quick peristaltic movement. Those experiments are viewed by him as *pathological* only, not physiological, in their results.

The subjects of Inflammation, Asphyxia, and Fever, are touched upon, so far as concerns their bearings upon the circulation of the blood. Inflammation is defined as "*a local lesion of nutrition, with concentric vascular excitement, resulting in exudation.*" Asphyxia is treated of in harmony with the view of Bichat, that oxygen is the normal excitant of the respiratory act, as well as of the pulmonary circulation; the adverse of the opinion suggested by the experiments and observations of Brown-Sequard. A brief discussion occurs of the recent ratiocinations and observations upon the subject of Fever, by Dr. H. T. Campbell, Virchow, Vogel, and E. Parkes; with the expression of the opinion that the condition of the bloodvessels must not be overlooked in the attempt to solve the difficult problem.

Lastly, a very careful examination is entered into of the principal theories of the Pulse, as held by Hunter, Sir C. Bell, Bichat, Young, Parry, Colt, and Arnott.

The general tenor of the argument of the essay (which has evidently been elaborated with great care), is stated in the following *resumé:*

"We conclude, as the summary result of the foregoing investigation—based, as we have seen, upon the combined facts of histology, analogy, experiment, and pathology,—that the most probable opinion or theory is (in opposition to most modern authorities, but in accordance with the views of Hunter and Sir Charles Bell, and partially with those of Carpenter), that, as all the arteries have a muscular coat, this is endowed, like the other muscular tissues of hollow viscera, with a power of alternating contraction and relaxation; that this contraction is exerted in immediate connection with, and successive upon, the beat of the heart; *the arterial systole thus combining with that of the ventricles to make up the pulse*: that the variation occurring in different parts of the circulation is to be accounted for to a considerable extent, although not entirely (a capillary power or nutritive affinity being also acknowledged), by the different degrees or kinds of *action* of the arteries; that the normal modes of stimulation by which these vessels are affected, are chiefly three: 1. That of *distension*, from the impulse of the heart; 2. The stimulus of *oxygen*, in the red corpuscles of the blood, by which all muscles are maintained in activity; and 3. The direct (*and reflex*) influence of the *nervous system*, by means of the arterial or vaso-motor nerves; lastly, it is by the sympathetic system that the principal control is maintained over the arteries, as well as over the heart; while they are subjected also to influences directly transferred or reflected from the cerebro-spinal axis, and to some which result from contact with the external world." P. 836.

In the statement, at the end of the volume, of the "Resolutions adopted at the Meeting at Detroit," an inadvertence appears to have occurred. The Resolution of Dr. Leidy, in regard to the future limitation of the award of prizes to essays of *original observation*, is there placed as having been adopted; whereas, according to the printed minutes, it was not adopted, but was referred to a committee (Drs. Leidy, Wood, and Meigs), to report upon next year.

The style of this volume of the Transactions certainly does great credit to the printers, as well as to the Committee of Publication. To our eye, ponderous though it be, they have succeeded in making it a handsomer volume than any one of its predecessors.

